# Probing the mechanisms for the selectivity and promiscuity of methyl parathion hydrolase

**DOI:** 10.1098/rsta.2016.0150

**Published:** 2016-11-13

**Authors:** Miha Purg, Anna Pabis, Florian Baier, Nobuhiko Tokuriki, Colin Jackson, Shina Caroline Lynn Kamerlin

**Affiliations:** 1 Science for Life Laboratory, Department of Cell and Molecular Biology, Uppsala University, BMC Box 596, Uppsala 75124, Sweden; 2 Michael Smith Laboratories, University of British Columbia, 2185 East Mall, Vancouver, BC, Canada V6T 1Z4; 3 Research School of Chemistry, Building 138, The Australian National University, Canberra, Australian Capital Territory 0200, Australia

**Keywords:** enzyme catalysis, catalytic promiscuity, metal selectivity, protein evolution, organophosphate hydrolysis, empirical valence bond

## Abstract

Diverse organophosphate hydrolases have convergently evolved the ability to hydrolyse man-made organophosphates. Thus, these enzymes are attractive model systems for studying the factors shaping enzyme functional evolution. Methyl parathion hydrolase (MPH) is an enzyme from the metallo-β-lactamase superfamily, which hydrolyses a wide range of organophosphate, aryl ester and lactone substrates. In addition, MPH demonstrates metal-ion-dependent selectivity patterns. The origins of this remain unclear, but are linked to open questions about the more general role of metal ions in functional evolution and divergence within enzyme superfamilies. Here, we present detailed mechanistic studies of the paraoxonase and arylesterase activities of MPH complexed with five different transition metal ions, and demonstrate that the hydrolysis reactions proceed via similar pathways and transition states. However, while it is possible to discern a clear structural origin for the selectivity between different *substrates*, the selectivity between different *metal ions* appears to lie instead in the distinct electrostatic properties of the metal ions themselves, which causes subtle changes in transition state geometries and metal–metal distances at the transition state rather than significant structural changes in the active site. While subtle, these differences can be significant for shaping the metal-ion-dependent activity patterns observed for this enzyme.

This article is part of the themed issue ‘Multiscale modelling at the physics–chemistry–biology interface’.

## 1. Introduction

Despite the classical image of enzymes as highly specific catalysts [1], recent studies have shown that many (if not even most) enzymes are catalytically promiscuous, facilitating at least one additional side reaction in addition to their native activity [2–4]. Such multifunctional enzymes provide effective starting points for the insertion of new catalytic activities, and there has, therefore, been an explosion of interest in harnessing catalytic promiscuity for enzyme design purposes [3,5]. In addition, promiscuous organophosphate hydrolases provide particularly interesting model systems for studying the factors shaping the evolution of enzyme function, as organophosphates have only been in widespread usage since the 1940s onwards, yet a broad range of enzymes from unrelated organisms, and with distinct overall protein folds, have been shown to have convergently evolved the ability to hydrolyse these compounds [6,7]. These enzymes can also have very different catalytic architectures and metal-ion dependencies, such as serum paraoxonase 1 (PON1), which uses a single catalytic calcium ion [8], and the bacterial phosphotriesterase (PTE), which has two zinc ions in its active site [9]. The distinct catalytic architectures, which evolved independently to catalyse the same reaction, provide an interesting model system to study the overall mechanistic requirements for enzyme function. In addition, there is great clinical interest in organophosphatases as potential biotherapeutics for the treatment of acute organophosphate poisoning [10,11].

**Figure 1.**
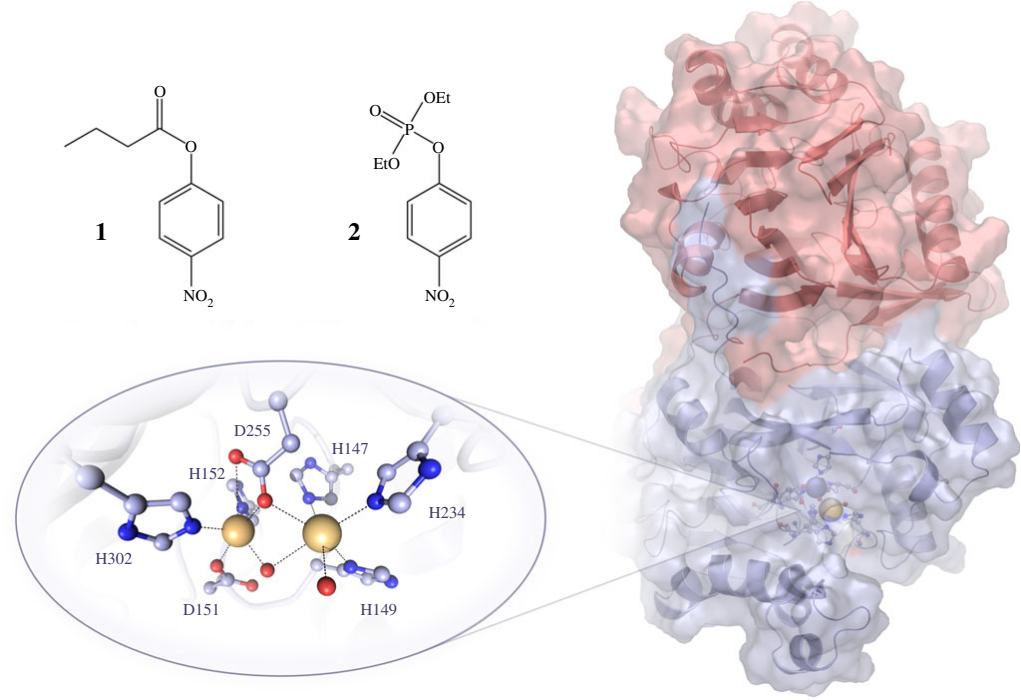
Overall structure of MPH, with a close-up of the key active site residues. Shown here are also the chemical structures of the two substrates of interest to this study, i.e. *p-*nitrophenyl butyrate (**1**) and paraoxon (**2**).

Here, we present a detailed computational study of the mechanisms and specificity patterns of the enzyme methyl parathion hydrolase (MPH), a member of the metallo-β-lactamase (MBL) superfamily [12–15], which has the overall structure and active site architecture shown in figure 1. MPH is particularly interesting in an evolutionary context, as it has only recently evolved the ability to hydrolyse a wide range of man-made organophosphates including the highly toxic pesticide methyl parathion [13] with a catalytic proficiency as high as *k*_cat_/*K*_M_ of 10^6^ M^−1^ s^−1^ [16]. In addition to this organophosphate hydrolase activity, MPH retains significant (presumably original/ancestral) arylesterase and lactonase activities [12,17], which make it an excellent model system to understand the fundamental features that allow an enzyme to diverge into an efficient organophosphatase. Following from this, as with many other phosphatases, MPH is a metalloenzyme with a typical binuclear active site (figure 1). Previous work has shown the most likely native metal ion to be Zn^2+^, but related MBLs can be activated or substituted with other metal ions such as Fe^2+^–Zn^2+^, Mn^2+^, Co^2+^ or Ni^2+^, among other possibilities [13–15,18]. We have recently screened the activity profiles of different metal-ion isoforms of five different members of the MBL superfamily, including MPH with six different divalent metals, namely Cd^2+^, Co^2+^, Fe^2+^, Mn^2+^, Ni^2+^ and Zn^2+^, and demonstrated interesting metal-ion-dependent specificity patterns, showing that the metal-ion preferences for the native and promiscuous activities are not necessarily correlated, and can be mutually exclusive [17]. This is significant, as several studies have demonstrated that some metal ions can alter the activity levels of a metalloenzyme towards non-native substrates [19–23], or, in the case of the aforementioned MBL superfamily members, reveal ‘cryptic promiscuous activities’ that were not observed with the presumably native metal ion [17]. In addition, often vastly different metal-ion requirements can be seen between otherwise functionally and evolutionarily related enzymes (e.g. among the different metallophosphatases in the alkaline phosphatase superfamily [24]). Therefore, a question remains about the role of metal ions in functional divergence within enzyme superfamilies, and the acquisition of new promiscuous activities.

Although there have been extensive biochemical (and more limited structural) studies of MPH [13–17,25], there have to date been no computational studies of the mechanisms of the hydrolytic activity of this enzyme towards any substrate, and the mechanistic conclusions that have been drawn about this system have mainly been through comparison with other related organophosphatases [7,13,14]. We recently developed force-field independent non-bonded parameters for a broad range of divalent metal ions [26] based on the original model of Åqvist & Warshel [27], and have used these parameters to study a range of biological systems [26,28–30]. We present herein a detailed empirical valence bond (EVB) study of the paraoxonase and arylesterase activities of MPH in complex with different divalent metal ions, providing a detailed mechanistic picture for both native and promiscuous activities, as well as insight into the origins of the metal-ion selectivity exhibited by this enzyme. We also demonstrate the key mechanistic differences between MPH and other organophosphate hydrolases with similar active site architectures.

## 2. Results and discussion

### (a) Mechanism of paraoxon hydrolysis by methyl parathion hydrolase

**Figure 2.**
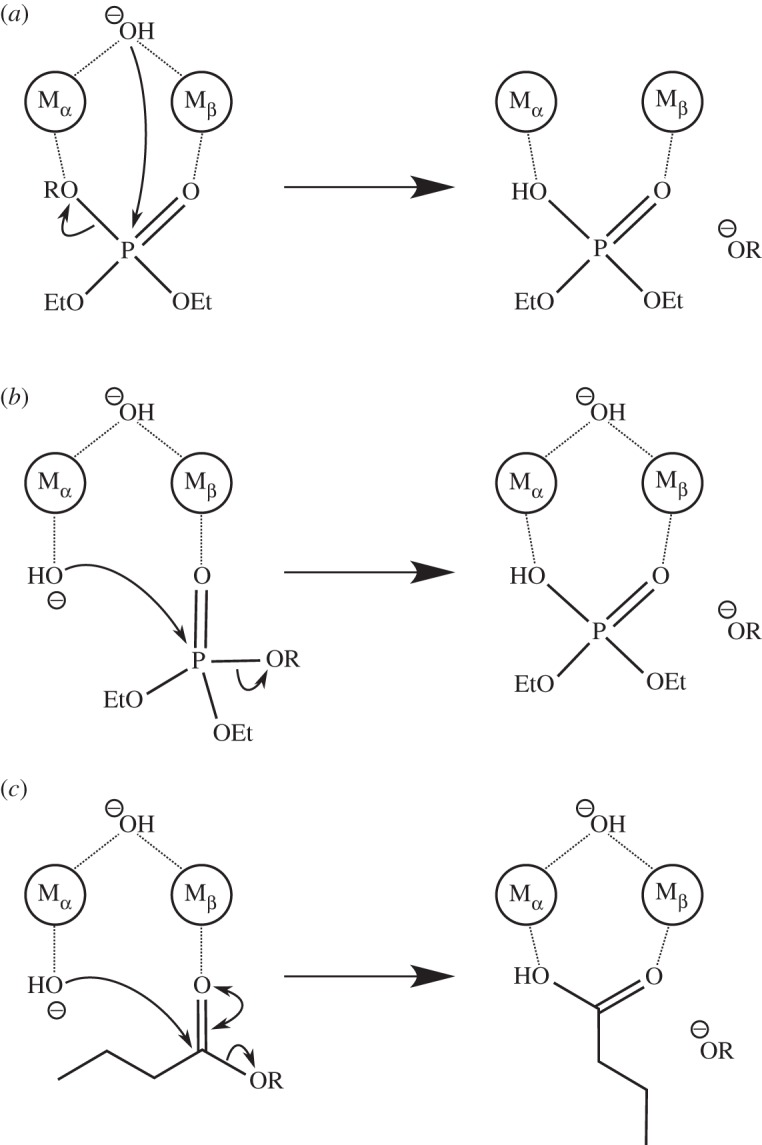
Comparison of different plausible mechanisms for the MPH-catalysed hydrolyses of (*a*,*b*) paraoxon and (*c*) *p*-nitrophenyl butyrate. OR in this case denotes the *p*-nitrophenyl leaving group.

Figure 2 shows a comparison of two viable catalytic mechanisms for paraoxon hydrolysis by MPH, based both on suggestions in the literature for analogous enzymes [9,31,32], as well as studies using model systems [33–35]. In particular, the first of these two mechanisms, involving a μ-bridging hydroxide ion as a nucleophile (that may or may not in turn be deprotonated by the side chain of D151) (figure 2*a*), is analogous to that proposed for the bacterial PTE [7,36,37], although other mechanisms have also been proposed [31,38,39]. The active sites of the two enzymes are very similar, and, in addition to possessing binuclear active sites, both enzymes have active sites comprised of three distinct hydrophobic pockets [7]. However, in addition to differences in metal-ion coordination geometries (where the metals in PTE adopt a trigonal bipyramidal or octahedral geometry and those in MPH are octahedrally coordinated), based on the shape of MPH's active site cavity, it is not possible for the substrate to bind in a position amenable for in-line attack by the bridging hydroxide ion (due to steric limitations). Therefore, we considered an alternative mechanism involving nucleophilic attack of a terminal hydroxide bound to the α-metal ion on a phosphate with monodentate coordination to the β-metal ion (figure 2*b*) through the P=O bond, with concomitant cleavage of the P–O bond to the aryl leaving group, in agreement with both experimental and computational studies of the alkaline hydrolysis of paraoxon [33–35,40,41], and studies of designed binuclear catalysts of phosphate hydrolysis reactions [42] and crystallographic studies of a related PTE from *Agriobacterium radiobacter* [31], as well as other metallophosphatases [43,44]. This was made feasible by manually placing paraoxon in a solvent-exposed pocket initially formed by the sidechains of R72, L67, L258, L273, F119 and F196 (the latter two of which have been suggested to be particularly important for MPH activity based on mutagenesis studies where truncation of these residues to alanine significantly abolished activity [13]). This resulted in an average P–O_nuc_ distance of 3.75 ± 0.24 Å and an average O_lg_–P–O_nuc_ angle of 160.1 ± 7.4° over the course of 40 ns of molecular dynamics (MD) equilibration (averages and standard deviations over all equilibration runs with all five metal ions), which is well positioned for in-line attack of the terminal hydroxide on the phosphate, as well as providing an exit route for the substrate after completion of the reaction. We note as an aside that for all metal ions studied here, upon equilibration, the D255 sidechain moved from a monodentate bridging position between the two metal ions as seen in the crystal structure to a bidentate position bridging both metal ions that is similar to that of the carbamylated lysine seen in crystal structures of PTE.

**Figure 3.**
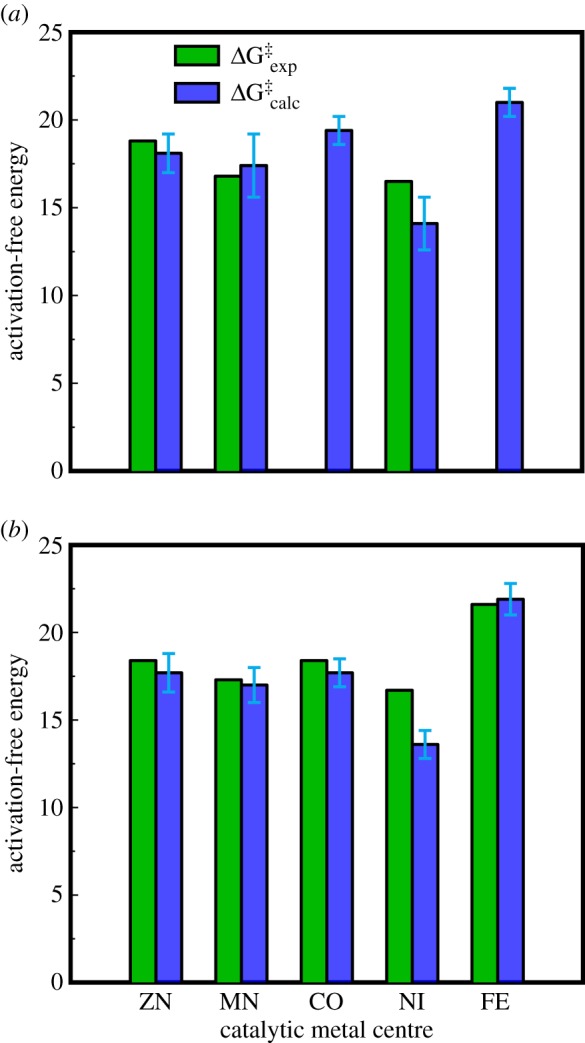
Comparison of the experimental (ΔG^‡^_exp_, green) and calculated (ΔG^‡^_calc_, blue) activation-free energies for the hydrolysis of (*a*) paraoxon and (*b*) *p*-nitrophenyl butyrate by MPH in complex with different metal ions. Error bars on the calculated values represent standard deviations calculated over 10 discrete EVB trajectories for each substrate and metal ion. The corresponding data are shown in electronic supplementary material, tables S1 and S2, and ΔG^‡^_exp_ was calculated from kinetic data presented in [17]. Note that in the two cases where experimental data is not presented in panel (*a*), it was not possible to obtain *k*_cat_ [17].

Following from this, we modelled the MPH-catalysed hydrolysis reaction proceeding through the terminal hydroxide mechanism in the presence of different metal ions using a simple two-state VB model, as illustrated in electronic supplementary material, figure S1, and the corresponding experimental and calculated energetics for each system are shown in figure 3 and electronic supplementary material, table S1 (experimental data based on values presented in [17]). From this figure and table, it can be seen that in the case of Zn^2+^, Mn^2+^ and Ni^2+^, for which *k*_cat_ values could be measured (thus providing an upper limit for the activation barrier to the chemical step), we are able to reproduce the relative trends in the three metals (i.e. Δg^‡^_Zn_ > Δg^‡^_Mn_ > Δg^‡^_Ni_), with reasonable agreement with the experimental values. Additionally, in the case of Co^2+^ and Fe^2+^ where *K*_M_ is so high (greater than 10 mM) that only *k*_cat_/*K*_M_ values are available, we obtain higher activation-free energies than for the three other metal ions, which is particularly relevant in the case of Fe^2+^, where the measured *k*_cat_/*K*_M_ for Fe^2+^ drops by 1000-fold compared with the most active metals, i.e. Mn^2+^ and Ni^2+^.

**Figure 4.**
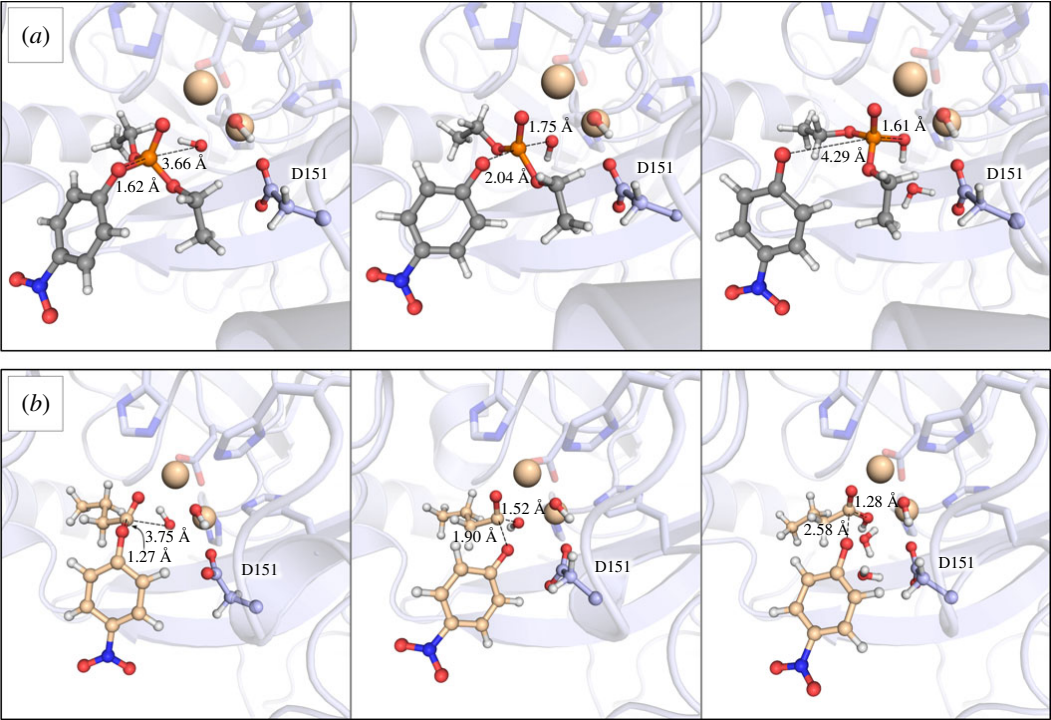
Representative structures of the Michaelis complexes, transition states and product states for the hydrolysis of (*a*) paraoxon and (*b*) *p*-nitrophenyl butyrate, in complex with two Zn^2+^ ions in the active site. The P(C)–O_nuc_ and P(C)–O_lg_ distances highlighted on each structure correspond to the average values shown in table 1. The catalytic metal centres, bridging and terminal hydroxide ions, substrate, and the side chain of D115 have been shown in ball and stick, and our octahedral dummy models as standard van der Waals spheres for clarity.

Following from this, figure 4 shows representative snapshots of the Michaelis complex transition state and product complex for MPH-catalysed paraoxon hydrolysis (with Zn^2+^ as the catalytic metal), and table 1 shows a comparison of the average P–O_nuc_, P–O_lg_ and metal–metal distance at the transition state averaged over all transition state frames for each metal ion. From these figures, it can be seen that the reaction proceeds through very geometrically similar tight transition states, as expected for phosphate triester hydrolysis based on studies with model compounds (for discussion, see e.g. [45,46] and references cited therein). In addition, the average metal–metal distance for the simulations with the different metal ions follows the trend expected from the differences in the ionic radii and metal aquo coordination distances of these metal ions as presented in [47] (see table 1 for the calculated values). Structurally, the transition state resembles a phosphorane intermediate, and already at the Michaelis complex the bulky ethyl sidechain of paraoxon has broken the hydrogen bond between the bridging hydroxide ion and the D151 sidechain (a structural perturbation that is not seen in the case of the simulations with the aryl ester, as discussed below). Finally, due to the valence bond states chosen to describe this process, our product state corresponds to a protonated diester bridging two metal ions, which is clearly a high-energy transient species. From figure 4*a*, it can be seen that, in the product state of the reaction, there is now a water molecule located between the OH group of the protonated phosphodiester (formerly the terminal hydroxide ion) and the sidechain of D151 (note that occasionally in our simulations, a second water molecule is involved in forming a network between the OH group and the D151 side chain). Therefore, it is highly likely that D151 will assist in deprotonating this OH group, presumably via these water molecules at a late stage in the reaction, as has been also suggested for PTE (for variations, see e.g. [7,36,37,39], although these studies assumed direct deprotonation from the corresponding Asp sidechain). However, as our calculated energetics are reasonable even without explicitly modelling this deprotonation step, we posit that it is a consequence of the formation of the protonated diester, rather than a contributor to the rate limiting step for this process.

**Table 1.** Comparison of the average P(C)–O_nuc_, P(C)–O_lg_ and metal–metal distances at the Michaelis complex, as well as the transition and products state (denoted RS, TS and PS, respectively) averaged over all RS, TS, PS frames for each metal ion. All distances are in Å. Values denote averages and standard deviations over 10 independent EVB trajectories for each system.

	paraoxon				*p*-nitrophenyl butyrate		
	P–O_nuc_				C–O_nuc_		
	RS	TS	PS		RS	TS	PS
Zn^2+^	3.66 ± 0.18	1.75 ± 0.05	1.61 ± 0.03	Zn^2+^	3.75 ± 0.28	1.52 ± 0.06	1.28 ± 0.04
Mn^2+^	3.97 ± 0.17	1.77 ± 0.06	1.61 ± 0.03	Mn^2+^	3.75 ± 0.24	1.55 ± 0.08	1.26 ± 0.03
Co^2+^	4.06 ± 0.21	1.76 ± 0.07	1.62 ± 0.02	Co^2+^	3.59 ± 0.23	1.51 ± 0.05	1.27 ± 0.03
Ni^2+^	3.75 ± 0.20	1.72 ± 0.06	1.62 ± 0.03	Ni^2+^	3.52 ± 0.27	1.56 ± 0.08	1.28 ± 0.04
Fe^2+^	3.89 ± 0.21	1.76 ± 0.06	1.61 ± 0.02	Fe^2+^	3.97 ± 0.17	1.57 ± 0.07	1.27 ± 0.03

	P–O_lg_				C–O_lg_		
	RS	TS	PS		RS	TS	PS
Zn^2+^	1.62 ± 0.03	2.04 ± 0.11	4.29 ± 0.41	Zn^2+^	1.27 ± 0.03	1.90 ± 0.10	2.58 ± 0.23
Mn^2+^	1.60 ± 0.03	1.95 ± 0.09	4.14 ± 0.30	Mn^2+^	1.26 ± 0.02	1.84 ± 0.11	3.01 ± 0.38
Co^2+^	1.61 ± 0.02	1.96 ± 0.09	4.01 ± 0.35	Co^2+^	1.28 ± 0.03	1.91 ± 0.10	2.82 ± 0.33
Ni^2+^	1.61 ± 0.03	2.04 ± 0.12	4.22 ± 0.31	Ni^2+^	1.27 ± 0.03	1.82 ± 0.09	3.01 ± 0.41
Fe^2+^	1.61 ± 0.03	2.00 ± 0.09	4.11 ± 0.34	Fe^2+^	1.27 ± 0.03	1.84 ± 0.09	2.95 ± 0.47

	M_α_–M_β_				M_α_–M_β_		
	RS	TS	PS		RS	TS	PS
Zn^2+^	3.75 ± 0.06	3.61 ± 0.06	3.64 ± 0.05	Zn^2+^	3.70 ± 0.09	3.58 ± 0.07	3.58 ± 0.05
Mn^2+^	3.88 ± 0.07	3.73 ± 0.05	3.79 ± 0.06	Mn^2+^	3.87 ± 0.08	3.72 ± 0.06	3.69 ± 0.05
Co^2+^	3.74 ± 0.07	3.61 ± 0.06	3.64 ± 0.06	Co^2+^	3.70 ± 0.05	3.58 ± 0.06	3.57 ± 0.06
Ni^2+^	3.68 ± 0.08	3.59 ± 0.06	3.52 ± 0.17	Ni^2+^	3.66 ± 0.08	3.60 ± 0.05	3.58 ± 0.04
Fe^2+^	3.76 ± 0.07	3.65 ± 0.07	3.69 ± 0.06	Fe^2+^	3.80 ± 0.06	3.66 ± 0.07	3.65 ± 0.06

### (b) Mechanism of arylester hydrolysis by methyl parathion hydrolase

A significant difference between phosphate and aryl ester hydrolysis is the preferred angle of attack, such that phosphate ester hydrolysis occurs primarily through an in-line nucleophilic displacement reaction, whereas for orbital overlap reasons, ester hydrolysis proceeds through a Bürgi–Dunitz trajectory with an angle of attack of at least 90°. While this does not rule out a mechanism involving nucleophilic attack by a terminal hydroxide ion similar to the model used for paraoxon hydrolysis, it does mean that the substrate needs to bind slightly differently in order to facilitate this (cf. the Michaelis complexes for paraoxon and *p*-nitrophenyl butyrate hydrolysis in figure 4, as well as the post-equilibration binding pockets for the two substrates shown in electronic supplementary material, figure S2). That is, in the case of the hydrolysis of *p*-nitrophenyl butyrate, the binding mode that aligns the substrate for optimal nucleophilic attack is in pockets formed by R37, L67, F119, H149, P150, F196, L258 and L273. This results in an average C–O_nuc_ distance of 3.64 ± 0.29 Å and an average O_nuc_–C=O angle of 81.4 ± 6.5° over the course of our initial MD equilibrations (averages and standard deviations over all equilibration runs with all five metal ions). Additionally, despite the differences in binding mode, we observe that after 40 ns of MD simulation, the ester groups of both substrates overlay almost perfectly on the β-metal. This has also been suggested for other organophosphatases, such as serum paraoxonase 1 (PON1), in the case of which the P(C)=O bonds of the chemically distinct phosphotriester and lactone substrates are activated by the catalytic Ca^2+^ ion in the same way [6]. Similarly, even though different residues are involved in substrate positioning and transition state stabilization for the two reactions, nevertheless, both interact with the metal ions in the MPH active site, which clearly plays an important role in the activation of the ester bond for hydrolysis.

Following from this, there has been substantial debate as to whether ester hydrolysis is a concerted process or a stepwise process involving a tetrahedral intermediate [48]. Based on computational studies of related reactions [49,50], and considering also the fact that *p*-nitrophenyl butyrate has a good leaving group, we have modelled the hydrolysis reaction as a single step concerted process as shown in figure 2, based on the valence bond states shown in electronic supplementary material figure S1. The resulting experimental and calculated energetics are shown in figure 3 and electronic supplementary material table S2. As can be seen from this table, we are able to both quantitatively and qualitatively reproduce the absolute and relative activation barriers for the hydrolysis of *p-*nitrophenyl butyrate in the presence of Zn^2+^, Co^2+^, Fe^2+^ and Mn^2+^ in the MPH active site. In the case of Ni^2+^, we underestimate the activation barrier compared to experimental data, although this was also the case in paraoxon hydrolysis and may be due to uncertainties in the experimental solvation-free energies that this metal ion has been parameterized to (see detailed discussion of this issue in [26,51,52]).

An interesting side observation is that in the case of the background reaction, as would be expected, the rate of *hydroxide* attack is significantly faster than the rate of water attack on arylesters, which tends to be generally quite low (in the range of 10^−6^ s^−1^, see the experimental data in [53–55]). In the case of *p*-nitrophenyl butyrate hydrolysis, based on experimental data for analogous compounds, one would expect the alkaline hydrolysis of this compound to have an activation barrier of approximately 15.8 kcal mol^−1^ [53]. By contrast, the water reaction is expected to have an activation-free energy closer to 26 kcal mol^−1^, again based on analogous compounds [56]. The activation-free energies measured for the MPH-catalysed hydrolysis of *p*-nitrophenyl butyrate are in the range of 17–22 kcal mol^−1^ (see electronic supplementary material table S2). Therefore, while the enzyme can significantly reduce the activation-free energy compared with the *water* reaction, presumably by generating a hydroxide nucleophile on the catalytic metal centre, the enzymatic deprotonation of water as a nucleophile (in the presence of metal ions) still does not allow the enzyme-catalysed reaction to match the hydroxide reaction. It increases our confidence in our calculations that we can quantitatively reproduce this difference (see the detailed methodology section in the electronic supplementary material information for further details of the calibration of our EVB calculations).

Additionally, as with paraoxon hydrolysis, the transition states are geometrically similar with all five metal ions with only slight differences in the C–O_lg_ distances to the leaving group, and the metal–metal distances follow a similar trend to that observed in the case of paraoxon (see table 1 for a distance overview). The biggest geometric change between the two substrates, apart from substrate positioning, is the fact that unlike in paraoxon hydrolysis, in this case the hydrogen bond between the bridging hydroxide ion and the D151 is maintained throughout our simulations and up to the transition state, but it is broken upon approaching the product complex, presumably again to facilitate deprotonation of the product by D151 through a chain of water molecules (see figure 4 for a representative Michaelis complex, transition state and product complex in the presence of Zn^2+^). However, while there are geometric changes between the two *substrates*, once again, and as can be seen from table 1, the differences between the *metal ions* are limited to the subtle changes one would expect from the differences in the ionic radii of these ions.

### (c) Origins of the metal-ion selectivity of methyl parathion hydrolase and its evolutionary implications

Having established that we can reproduce the metal-ion selectivity of MPH with both the organophosphate and aryl ester substrates, the question of the origin of this observed effect still remains. One of the key roles of metal ions in MBL superfamily members is to activate a metal-ion-bound hydroxide ion for nucleophilic attack [3,17,57]. Therefore, one would assume that at least part of the observed differences in activity can be linked to differences in hydroxide p*K*_a_ when different metal ions are present in the MPH active site. However, the p*K*_a_s of the metal aquo complexes of the metal ions being considered in this work are very similar, with an average p*K*_a_ of 9.7 ± 0.6 across all five metal ions. Therefore, one would expect at most a contribution of 1 kcal mol^−1^ to the differences in activation barrier from differences in nucleophile p*K*_a_, whereas the actual differences in activation barrier that are observed both experimentally and computationally (figure 3; electronic supplementary material, tables S1 and S2) can be as high as 4 kcal mol^−1^ between the different metals. Thus, differences in nucleophile p*K*_a_ are not enough to explain the origins of the metal-ion selectivity of MPH, especially taking into account the fact that we are able to reproduce both qualitative and quantitative trends between different metal ions using a classical model that does not explicitly describe ligand-to-metal charge transfer.

The second catalytic role of the metal ions in the MPH active site is to act as Lewis acids, thus stabilizing the ground and transition states of the reactions involved [3,17,57]. From table 1, it can be seen that for both the neutral organophosphate and the anionic aryl ester, the transition states are geometrically very similar between the different metal ions. Following from this, despite some quantitative differences (due, in particular, to the flexibility in our simulations of a loop comprising residues A115–G124; electronic supplementary material, figure S3), the comparison of the electrostatic contributions of each amino acid in the protein to the overall calculated free energy shows similar contributions towards the stabilization of each transition state for the *same* substrate for the simulations with different metal ions (electronic supplementary material, figure S4). In addition, there are some quantitative differences between the two substrates due to the differences in binding mode and inherent differences in their chemistry, even though largely the same residues are involved in stabilization of both transition states.

The main origin of the activity differences between the various metal ions appears, however, to be due to the differences in the solvation-free energies between the different metal ions (which is inherent to the parameterization of the different metal ions [26]), as this is the main factor that is different between our simulations. These differences in ΔΔ*G*_solv_ will, in turn, affect how well each metal ion can stabilize the metal-ion-bound transition state for each reaction. In addition, the subtle differences between the calculated metal–metal distances for different metal ions (table 1) not only affect the positioning of the substrate and the nucleophile relative to each other in the Michaelis complex, but also the corresponding transition state geometries and thus the electrostatic stabilization of the different transition states. This suggests, therefore, that the metal-ion-dependent changes in activity observed for the hydrolysis of the two substrates by MPH is a purely electrostatic effect that is due to the inherent properties of each metal ion, rather than any radical changes in the active site. This is further supported by the observation that despite the significant differences in the positioning of both substrates in the active site, and thus the residues they interact with during the chemical reaction, nevertheless the same trend in metal-ion preferences is observed for both paraoxon and *p*-nitrophenyl butyrate.

In the case of the two substrates studied here, the requirements for efficient catalysis of their hydrolytic reactions are quite similar, and both reactions are already quite fast in the absence of the enzyme, when compared with other phosphoryl transfer reactions which can have half-lives in the millions of years [58]. Therefore, significant rate accelerations are obtained simply through positioning effects by the metal ions and activation of the ester bond of each substrate, as well as through generation of a metal-bound hydroxide nucleophile. Based on this, it is perhaps unsurprising that both substrates are easily catalysed by all five metal ions studied in this work. However, in the case of reactions which are far more difficult to catalyse, these subtle geometric and electrostatic changes can make the difference between whether a promiscuous activity is observed or not. For example, we also measured the rates of bis-*p*-nitrophenyl phosphate diester hydrolysis by MPH [17], which is a substantially more difficult reaction to catalyse (with a background rate of 6.3 × 10^−8^ s^−1^ for the spontaneous reaction in aqueous solution [59]), and could observe only diesterase activity in MPH in the presence of Ni^2+^ ions (and even this was very weak) [17]. Ni^2+^ is also the metal ion with the largest solvation-free energy of the metal ions studied in this work, and is thus the metal with the greatest ability to stabilize the phosphodiester transition state.

## 3. Overview and conclusion

In this work, we have provided the first detailed mechanistic study of the hydrolysis of representative organophosphate and aryl ester substrates by MPH, an organophosphatase from the MBL superfamily. We demonstrate that, in both cases, the reaction appears to proceed through nucleophilic attack by a terminal hydroxide ion. Following from this, the large active site volume of MPH allows for the enzyme to accommodate the geometric constraints for in-line nucleophilic attack on the organophosphate and an approximately 81° angle of attack on the aryl ester, even though this requires very different binding modes for the two substrates, resulting also in transition state stabilization from different active site residues. We have also examined the metal-ion-dependent activity patterns of MPH with five different divalent transition metal ions, namely Co^2+^, Fe^2+^, Mn^2+^, Ni^2+^ and Zn^2+^. We are able to reproduce experimental metal-ion-dependent activity patterns for both substrates [17], and demonstrate that the origin of this effect appears to be primarily differences in the electrostatic properties of the metals themselves, coupled with very subtle changes in substrate and transition state geometries with the different metal ions which affect charge distributions at the transition state and corresponding transition state stabilization, rather than large rearrangements of metal-ion coordination or active site architecture. As also shown in [17], while subtle, these differences can nevertheless be sufficient to make the difference between whether a cryptic promiscuous activity is exposed with a particular metal ion or not. Therefore, substrate-activity profiles can be controlled through judicious selection of different metal ions in the catalytic centre. Obtaining a better understanding of the molecular origin for these changes and how they can be controlled is therefore important for not only understanding the factors shaping the evolution of biological catalysts, but also providing a blueprint for the rational engineering of improved organometallic catalysts for phosphoryl transfer reactions modelled on enzyme active sites.

## 4. Methodology

The starting point for our simulations was the 2.4 Å structure of MPH from *Pseudomonas* sp WBC-3 (PDB ID: 1P9E) [13]. We re-refined this structure as described in the electronic supplementary material. The MPH-catalysed hydrolysis of both paraoxon and *p*-nitrophenyl butyrate were then studied by means of the EVB approach [60,61]. The calculations were based on the aforementioned crystal structure of MPH with manually docked substrates and the Zn^2+^/Cd^2+^ originally present in the structure replaced with either of two Co^2+^, Fe^2+^, Ni^2+^, Mn^2+^ or Zn^2+^ ions. The structures were equilibrated by performing an initial 40 ns MD simulation and then subjected to the standard free energy perturbation/umbrella sampling procedure [60–63] to determine free energy profiles for each reaction in the presence of different metal ions. The EVB simulations were performed in 51 mapping windows of 200 ps in length each, using 10 different starting structures. These were obtained by performing an additional 200 ps of MD simulations at the end of our initial 40 ns using 10 different random velocities, and using the resulting structures as starting points for our subsequent EVB simulations, leading to a total of 102 ns of simulation time per system. All calculations were performed using the Q simulation package [64] and the OPLS-AA force field [65], and the different metal centres were described using octahedral dummy models with the parameters described in previous work [26,27]. A more detailed methodology section is available as the electronic supplementary material, which also includes all EVB parameters used in this study.

## Supplementary Material

Kamerlin_ESI.pdf

## Data accessibility

The datasets supporting this article have been uploaded as part of the electronic supplementary material.
