# Supplementary material for: Probing the mechanisms for the selectivity and promiscuity of methyl parathion hydrolase
Source: Philos Trans A Math Phys Eng Sci. 2016 Nov 13;374(2080):20160150. doi: 10.1098/rsta.2016.0150 (PMC5052733; doi:10.1098/rsta.2016.0150)
Supplement: Kamerlin_ESI.pdf [file rsta20160150supp1.pdf]

Detailed Methodology

As with our other recent mechanistic studies of phosphoryl transfer transfer reactions [1-3], here, our methodology of choice was again the empirical valence bond (EVB) approach of Warshel and coworkers [4, 5]. This is a fully classical approach based on empirical force fields, which describes chemical reactivity within a quantum mechanical framework based on using a valence bond description of a reacting system [6]. Its broad applicability to the study of enzyme-catalyzed reactions in general and phosphoryl transfer reactions in particular is well-documented [1-3, 7], and it's primary advantages are that well-parameterized force fields carry a tremendous amount of chemical information, allowing one to describe chemical reactivity in physically meaningful fashion, while the fact that it is a classical approach means that it is possible to sample the environment on timescales that are currently inaccessible to higher-level (or even semi-empirical) quantum chemical approaches. Another advantage of the EVB approach is that it uses as its reaction coordinate the energy difference between the different diabatic states. As such, it uses what is essentially a non-geometric reaction coordinate, thus projecting the complex multidimensional reaction space onto a single reaction coordinate. This allows all relevant conformational changes (including active site reorganization) to be included in the EVB reaction coordinate.

The crystal structure of MPH at 2.4Å resolution and without a substrate bound was obtained from the Protein Data Bank [8, 9] (PDB ID: 19PE [10]). Although the structure was originally modelled with two zinc ions in the active site, inspection of electron density maps indicated that the metal ion at the more solvent exposed position (coordinated by His147, His149, His234) had greater electron density than would be expected from a zinc ion, and was replaced by a cadmium ion and re-refined. This led to an improved fit to the electron density (**Supplementary Figure 5**). The greater size of the cadmium at the more solvent exposed position would favour the monodentate coordination mode of Asp255. However, when replaced by a zinc ion, as in the MD simulations, the reduced size of the zinc ion would allow rearrangement of this side chain to a bidentate coordination mode, which was indeed observed to occur in the MD simulations when a Zn^{2+}-Zn^{2+} active site was used. A similar bidentate coordination of two metal ions by a carboxylic acid group is observed in a functionally related protein, the bacterial phopshotriesterase [11].

All molecular dynamics and EVB simulations in this work were performed using the Q simulation package [12] and the OPLS-AA force field [13]. OPLS-AA compatible force field parameters for paraoxon and *p*-nitrophenyl butyrate were generated using MacroModel version 10.3 (Schrödinger LLC, v. 2014-1) [14] and are provided as **Supporting Information**. The corresponding partial charges for these compounds were calculating using the standard RESP procedure [15], with the use of Antechamber [16] (AmberTools 12) [17] and Gaussian09 (Revision C.01 [18]). All simulations were performed by solvating the system in a spherical droplet with a radius of 20Å centred on the μ-bridging hydroxide ion of Chain A, and subject to surface-constrained all-atom solvent (SCAAS)

© The Authors under the terms of the Creative Commons Attribution License http://creativecommons.org/licenses/by/3.0/, which permits unrestricted use, provided the original author and source are credited.

boundary conditions [19]. All crystallographic water molecules from Chain A were retained for the simulations, and they were further complemented with TIP3P water molecules [20] within the 20Å sphere. With the exception of our initial molecular dynamics (MD) equilibration (see below), all protein atoms and water molecules within 85% of the sphere were allowed to move freely with no restraints, atoms in the last 15% of the sphere were subject to 10 kcal mol^{-1} $Å^{-2}$ positional restraints, and all atoms outside this sphere were subjected to 200 kcal mol^{-1} $Å^{-2}$ positional restraints to keep them to their crystallographic positions (see also our previous work [1, 2, 21]). With the exception of the very initial equilibration stage, all solvent molecules were constrained using the SHAKE algorithm [22].

Protonation states of all ionisable residues in the protein were determined based on empirical screening using PROPKA 3.1 [23, 24] as well as, in the case of histidine side chains, by visual inspection of the surrounding hydrogen bonding patterns. All residues in the inner 85% of the simulation sphere were protonated accordingly (see **Supplementary Table 3** for a list of ionized residues in the simulation) and all ionizable residues outside of this region were kept in their uncharged forms to avoid simulation artefacts that would be introduced by having charged residues outside the explicit simulation sphere. The nucleophile for the hydrolysis of both paraoxon and *p*-nitrophenyl butyrate hydrolysis was modelled as a hydroxide ion placed on the α-metal, and both the bridging and terminal hydroxide ions were modeled using partial charges of -1.1e and +0.1e on the oxygen and hydrogen atoms of the hydroxide ion respectively. The Zn^{2+}/Cd^{2+} of Chain A were then replaced with either of two Co^{2+}, Fe^{2+}, Ni^{2+}, Mn^{2+} or Zn^{2+} ions, described using Åqvist and Warshel's octahedral dummy model [25] for improved system stability, based on the parameters presented in ref. [26] (we have recently applied this model successfully to study several biological systems). This model allowed us to capture the main electrostatic properties of the different metal ions, as well as maintaining structural stability without the need of artificial bonds or restraints on the metal ions. Such a flexible non-bonded model allows for changes in metal-ligand interactions during the reaction coordinate to be captured (which is crucial for modeling the chemical reaction step in a meaningful way). The two metal ions present in Chain B were replaced with spherical dummy atoms with no charge or van der Waals parameters on them, again in order to avoid simulation artefacts arising from having charged residues or heteroatoms outside the explicit simulation sphere. However, as with other atoms outside the explicit sphere these metal ions were also subject to 200 kcal mol^{-1} $Å^{-2}$ positional restraints thus preventing the Chain B active site from collapsing during our simulations. Finally, the two substrates of interest to this work were then manually placed into the active site of Chain A, in such a way as to optimally position their scissile bonds for in-line attack or attack from a Bürgi-Dunitz angle [27] of 160.1 ± 7.4° and 81.4 ± 6.5° in the case of paraoxon and *p*-nitrophenyl butyrate respectively.

All systems were gradually heated from 1 to 300 K over a 60 ps timescale, while applying a 200 kcal mol^{-1} $Å^{-2}$ harmonic restraint on all the solute heavy atoms, in order to restraint them to their crystallographic positions. This both allowed the solvent to equilibrate around the solute as well as the removal of bad contacts in the initial placement of the hydrogen atoms. The system was then cooled to 5K for another 60 ps of simulation time, while gradually reducing the harmonic restraint from 200

© The Authors under the terms of the Creative Commons Attribution License http://creativecommons.org/licenses/by/3.0/, which permits unrestricted use, provided the original author and source are credited.

kcal mol^{-1} Å^{-2} on all solute heavy atoms to 0.5 kcal mol^{-1} Å^{-2} on only the substrate and terminal hydroxide ion (not including the restraints described above on atoms at the boundary of the simulation sphere and those outside it), and then reheated back to 300 K for 90 ps of simulation time, The initial system equilibration was then completed by performing a 40 ns molecular dynamics simulation at 300K, retaining only the weaker 0.5 kcal mol^{-1} Å^{-2} restraint on the reacting atoms in order to keep them within the simulation sphere (see **Supplementary Figure 6** for the C_α RMSDs). This equilibrated structure then formed the starting point of our subsequent EVB simulations, which were performed using the standard EVB free energy perturbation/umbrella sampling (EVB-FEP/US) procedure described in detail in refs. [4-7], and based on the valence bond structure described in **Supplementary Figure 1**. Here, as a first step, we performed an additional 200 ps of molecular dynamics simulations on the 40 ns equilibrated structure for each system using ten different initial starting velocities (obtained by using different random seeds for each simulation), in order to generate ten new starting structures for our subsequent EVB simulations. The EVB simulations themselves were then performed in 51 windows of 200ps in length, retaining the 0.5 kcal mol^{-1} Å^{-2} restraint on the reacting region, and resulting in a total simulation time of 10.2 ns of simulation time per trajectory, 102 ns of simulation time per system, and 1.02 μs of simulation time across all systems. All MD and EVB simulations were performed using the leap-frog integrator algorithm with an integration time step 1 fs, and a 10Å cutoff for calculating all non-bonded interactions, except for those involving atoms in the EVB region which were subjected to a 99Å cut-off (essentially no cut-off). Long-range electrostatic interactions beyond the cutoff were treated with the local reaction field method (LRF) [28] and the temperature of the system was regulated with the Berendsen thermostat [29] using a 100 fs bath relaxation time.

Finally, central to the EVB philosophy is the use of a well-calibrated reference state, which is usually either the uncatalyzed reaction in aqueous solution, or the wild-type enzyme relative to the set of mutants [4-7]. The coupling between the EVB parabola and their relative positions are then adjusted to reproduce either existing experimental data or high-level quantum chemical calculations, and the corresponding parameters are then used *unchanged* in all subsequent simulations of that reaction in order to facilitate direct comparison between the simulations. In the present case, our reference state for the MPH-catalysed hydrolysis of paraoxon and *p*-nitrophenyl butyrate was the corresponding hydrolysis using a hydroxide nucleophile in aqueous solution, and, fortunately, both reactions have been extensively experimentally characterized [30-33], and thus there is excellent data available based on which to calibrate the background reaction. Hydroxide attack on paraoxon and *p*-nitrophenyl butyrate were modelled using the exact same equilibration and EVB protocol as for the corresponding enzyme-catalysed reaction, with the exception of a shorter 10ns initial equilibration for each substrate, and a larger 1 kcal mol^{-1} Å^{-2} positional restraint on the reacting atoms due to the greater conformational flexibility in aqueous solution. The EVB parabola were then fit to reproduce activation free energies of 20.1 and 15.8 kcal mol^{-1} for paraoxon and *p*-nitrophenyl butyrate hydrolysis respectively, based on experimental data provided in ref. [30] (and by analogy to *p*-nitrophenyl

© The Authors under the terms of the Creative Commons Attribution License http://creativecommons.org/licenses/by/3.0/, which permits unrestricted use, provided the original author and source are credited.

acetate in the case of the arylester), and a reaction free energy of -10.6 kcal mol^{-1}, in analogy to ATP and acetyl phosphate hydrolysis (see discussion in the Supporting Information of ref. [2], note also that the precise reaction free energy used is less important in modeling a 2-step reaction as it represents a standard energetic shift of the parabolas throughout). The corresponding EVB parameters were then used unchanged in our EVB simulations of all corresponding enzyme-catalyzed reactions for each substrate, and all parameters necessary to reproduce our work have been provided as **Supplementary Tables 4-10**.

© The Authors under the terms of the Creative Commons Attribution License http://creativecommons.org/licenses/by/3.0/, which permits unrestricted use, provided the original author and source are credited.

Supplementary material for M. Purg, A. Pabis, F. Baier, N. Tokuriki, C. Jackson and S. C. L. Kamerlin, 2016, Probing the Mechanisms for the Selectivity and Promiscuity of Methyl Parathion Hydrolase, *Phil. Trans. R. Soc. A.* doi: 10.1098/rsta.[paper ID in form xxxx.xxxx e.g. 10.1098/rsta.2014.0049]

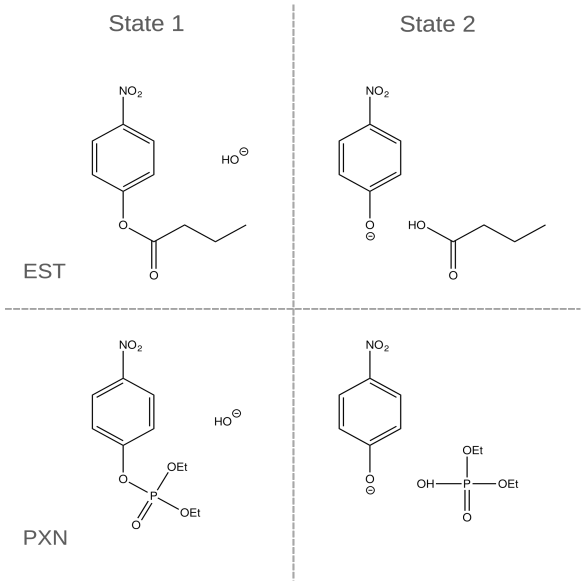

Supplementary Figure 1: Valence bond states used in our EVB calculations to describe the hydrolysis of paraoxon and *p*-nitrophenyl butyrate. States I and II refer to the reactant and product states for hydroxide attack on each of these compounds, respectively.

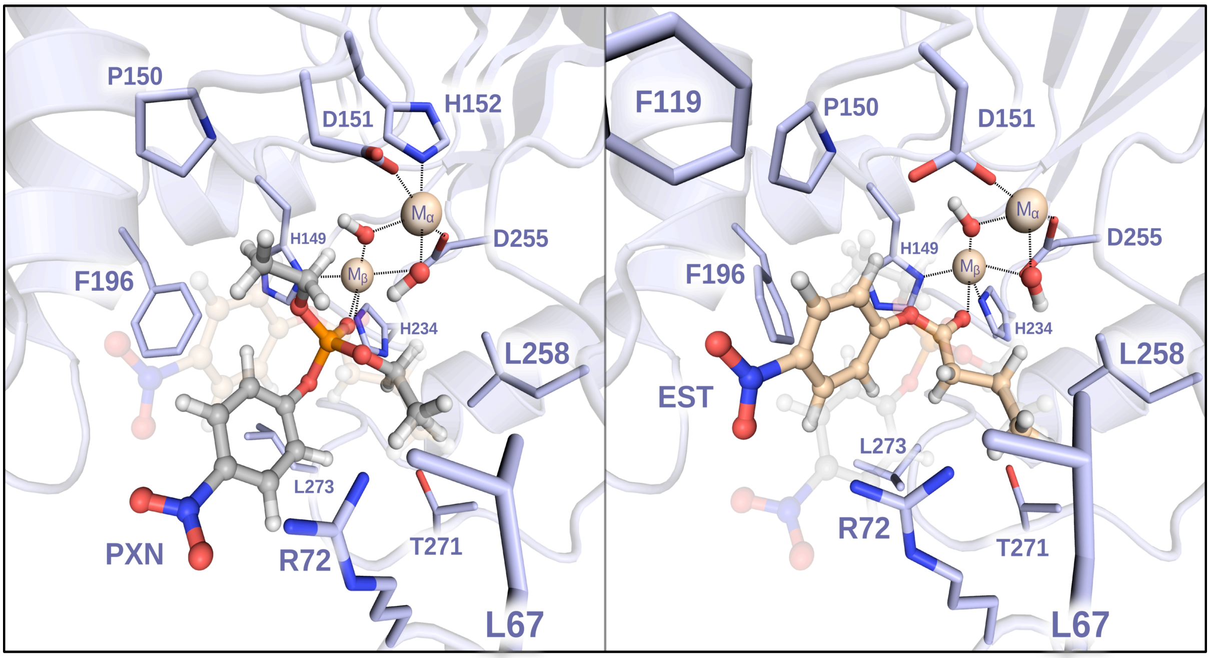

Supplementary Figure 2: A comparison of the binding pockets for (**A**) paraoxon and (**B**) *p*-nitrophenyl butyrate in the active site of MPH in complex with two catalytic Zn^{2+} ions, after 40 ns of molecular dynamics equilibration as described in the Methodology section. Note that our octahedral dummy models have been shown as standard van der Waals spheres for clarity.

© The Authors under the terms of the Creative Commons Attribution License http://creativecommons.org/licenses/by/3.0/, which permits unrestricted use, provided the original author and source are credited.

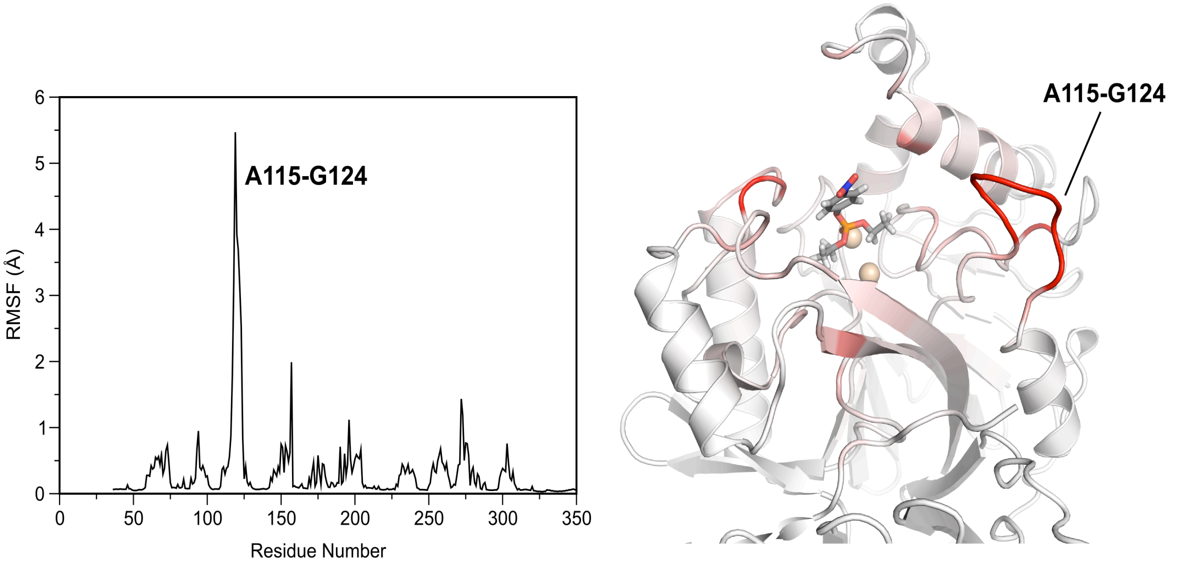

Supplementary Figure 3: (**Left**) Root mean square fluctuations (RMSF) of C_α atoms of MPH in complex with paraoxon and two catalytic Zn^{2+} ions. (**Right**) Structure of MPH colour coded according to calculated RMSF, with increasing intensity of red corresponding to higher RMSF. As can be seen from this figure, the loop formed by residues A115-G124 is considerably more flexible than the rest of the protein and can take many different conformations during the simulations; however, we were unable to see any correlation between loop position and calculated activation free energy.

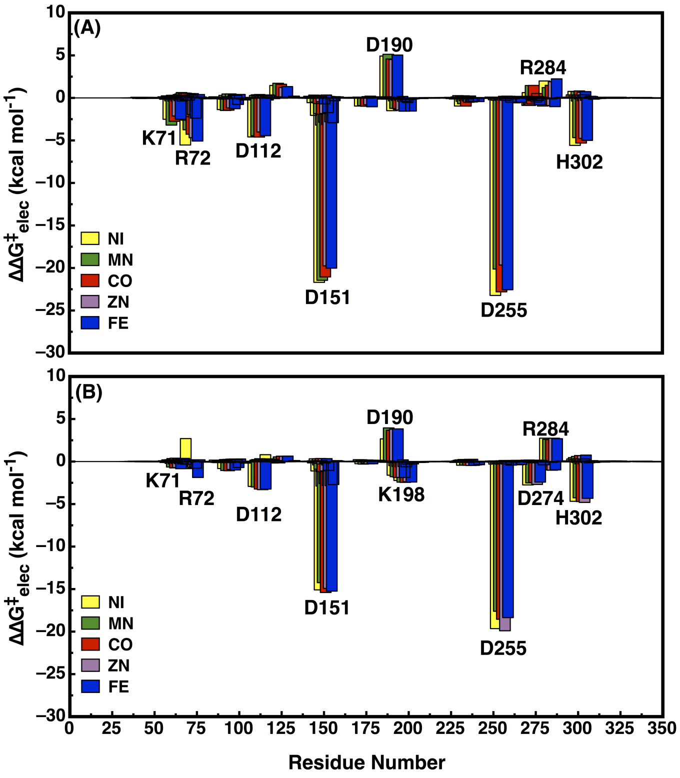

Supplementary Figure 4: A comparison of the electrostatic contribution of key residues to the calculated activation barrier for the hydrolysis of (**A**) paraoxon and (**B**) *p*-nitrophenyl butyrate by MPH in complex with five different metals ions. All calculations were performed by post-processing our EVB trajectories using the linear response approximation, as described in *e.g.* [21, 34].

© The Authors under the terms of the Creative Commons Attribution License http://creativecommons.org/licenses/by/3.0/, which permits unrestricted use, provided the original author and source are credited.

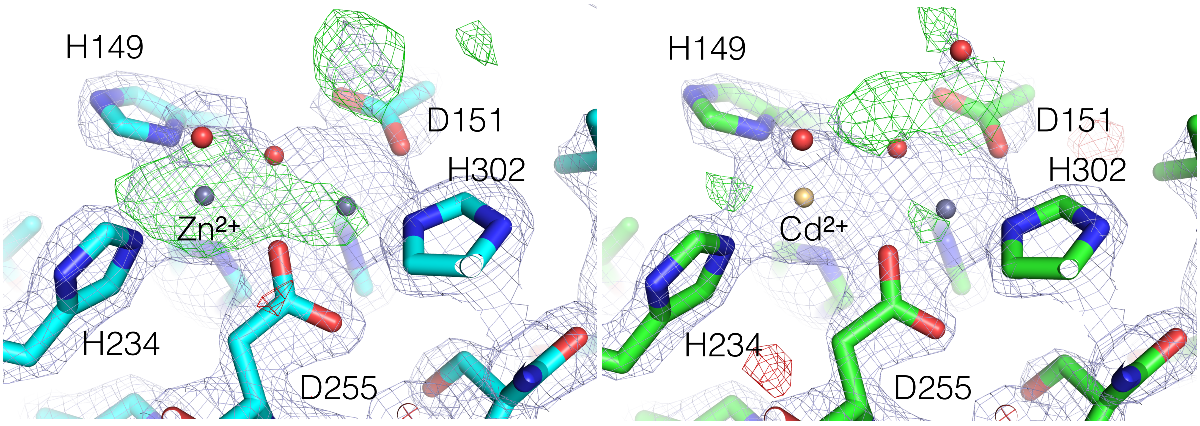

Supplementary Figure 5: The active site of MPH (PDB ID: 19PE) refined with Zn^{2+}-Zn^{2+} (left) and Zn^{2+}-Cd^{2+} (right) active sites. 2mFo-dFc electron density, contoured at 2 σ is shown in blue mesh, mFo-dFc difference density is shown in green (+3 σ) and red (-3 σ) electron density. The better fit of Cd^{2+} to the electron density is evident.

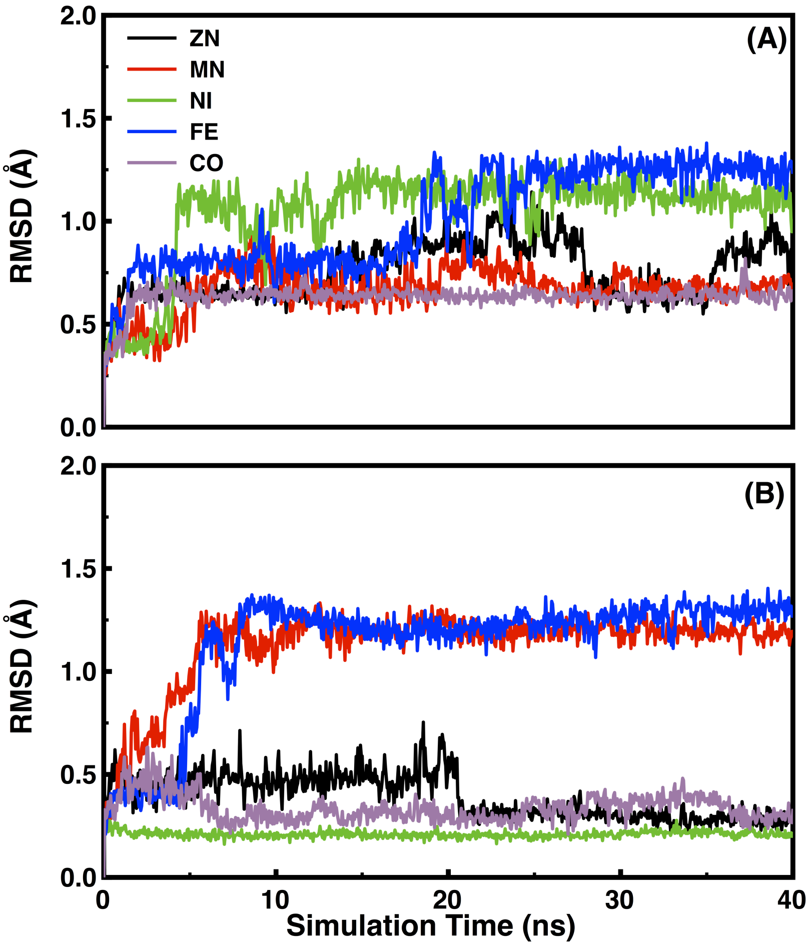

Supplementary Figure 6: Time evolution of the root-mean-square deviation (RMSD) of the C_α atoms of MPH during molecular dynamics simulations in the presence of different metal ions and in complex with (**A**) paraoxon and (**B**) *p*-nitrophenyl butyrate.

© The Authors under the terms of the Creative Commons Attribution License http://creativecommons.org/licenses/by/3.0/, which permits unrestricted use, provided the original author and source are credited.

	Experimental				Calculated	
	k_{cat} (s^{-1})	K_M (μM)	k_{cat}/K_M (M^{-1} s^{-1})	$\Delta G^‡$ (kcal/mol)	$\Delta G^‡$ (kcal/mol)	ΔG_0 (kcal/mol)
Zn^{2+}	0.1 ± 0.006	2200 ± 250	5.3 x 10^1	18.8	18.1 ± 1.1	-3.0 ± 1.4
Mn^{2+}	3.2 ± 0.1	1100 ± 80	2.9 x 10^3	16.8	17.4 ± 1.8	2.4 ± 1.3
Co^{2+}	n.d.	>10 000	1.9 x 10^2	n.d.	19.4 ± 0.8	2.7 ± 1.5
Ni^{2+}	4.8 ± 0.3	1300 ± 170	3.6 x 10^3	16.5	14.1 ± 1.5	-9.2 ± 1.7
Fe^{2+}	n.d.	>10 000	1.0 x 10^0	n.d.	21.0 ± 0.8	1.9 ± 1.0

Supplementary Table 1: Summary of the kinetic parameters and calculated energetics for the hydrolysis of paraoxon catalysed by MPH with various metal ions present in the active site. Experimental data are taken directly or based on values presented in ref. [35]. Calculated values represent averages and standard deviations over 10 independent EVB trajectories (see the Methodology section).

	Experimental				Calculated	
	k_{cat} (s^{-1})	K_M (μM)	k_{cat}/K_M (M^{-1} s^{-1})	$\Delta G^‡$ (kcal/mol)	$\Delta G^‡$ (kcal/mol)	ΔG_0 (kcal/mol)
Zn^{2+}	0.2 ± 0.01	>10 000	1.8 x 10^1	18.4	17.7 ± 1.1	4.2 ± 1.2
Mn^{2+}	1.3 ± 0.02	2500 ± 80	5.4 x 10^2	17.3	17.0 ± 1.0	2.7 ± 1.3
Co^{2+}	0.2 ± 0.01	1600 ± 180	1.4 x 10^2	18.4	17.7± 0.8	1.7 ± 1.2
Ni^{2+}	3.7 ± 0.09	2200 ± 100	1.7 x 10^3	16.7	13.6 ± 0.8	1.2 ± 0.8
Fe^{2+}	0.001 ± 0.0001	900 ± 210	1.3 x 10^0	21.6	21.9 ± 0.9	9.0 ± 1.0

Supplementary Table 2: Summary of the kinetic parameters and calculated energetics for the hydrolysis of *p*-nitrophenyl butyrate catalysed by MPH with various metal ions present in the active site. Experimental data are taken directly or based on values presented in ref. [35]. Calculated values represent averages and standard deviations over 10 independent EVB trajectories (see the Methodology section).

© The Authors under the terms of the Creative Commons Attribution License http://creativecommons.org/licenses/by/3.0/, which permits unrestricted use, provided the original author and source are credited.

Residue	Residue number
**Asp**	62, 112, 151, 190, 255, 274, 276
**Glu**	94, 175, 283
**Lys**	71, 174, 194, 198
**Arg**	72, 125, 284
**His-δ**	147, 152, 232, 234, 238, 302, 310
**His-ε**	149

Supplementary Table 3: List of residues that were ionized in the simulations, and protonation patterns of histidines. All other residues fell outside our mobile region and were thus kept in their unionized forms during the simulation to avoid having charged residues in the excluded region of our simulations (see also the Methodology section of the main text). Note that some of the ionized/unionized residues fell near the border of the simulation. Here, we adjusted the ionization state based on how close residues fell to the interface between the mobile and restrained regions, in such a way as to keep the overall system charge neutral.

© The Authors under the terms of the Creative Commons Attribution License http://creativecommons.org/licenses/by/3.0/, which permits unrestricted use, provided the original author and source are credited.

Supplementary material for M. Purg, A. Pabis, F. Baier, N. Tokuriki, C. Jackson and S. C. L. Kamerlin, 2016, Probing the Mechanisms for the Selectivity and Promiscuity of Methyl Parathion Hydrolase, *Phil. Trans. R. Soc. A.* doi: 10.1098/rsta.[paper ID in form xxxx.xxxx e.g. 10.1098/rsta.2014.0049]

Overview of EVB paramaters

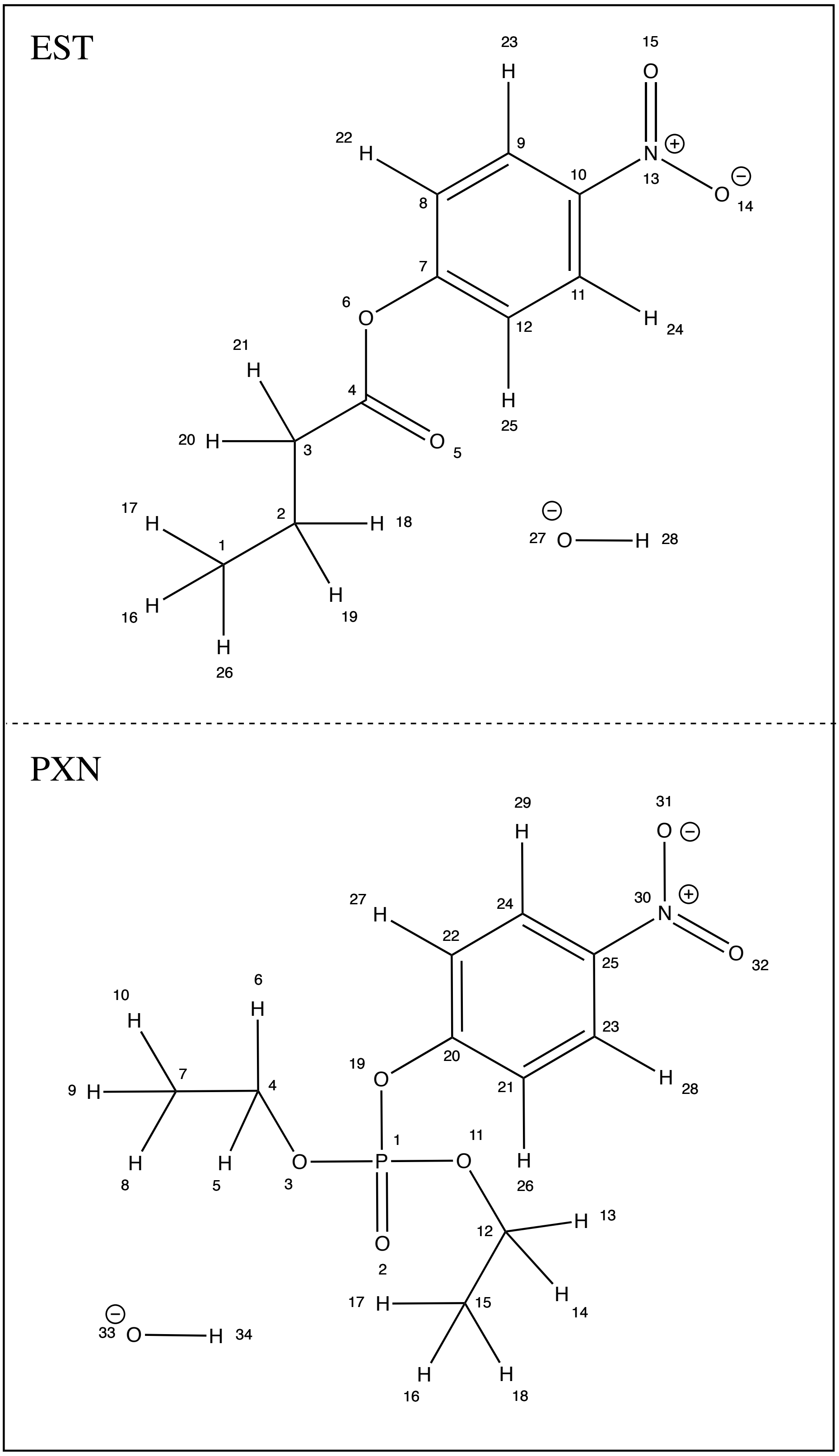

Supplementary Figure 7: Numbering of atoms constituting the reacting part of the system for the reaction of *p*-nitrophenyl butyrate (EST) and paraoxon (PXN).

© The Authors under the terms of the Creative Commons Attribution License http://creativecommons.org/licenses/by/3.0/, which permits unrestricted use, provided the original author and source are credited.

Van der Waals parameters used for the atoms in the reacting part of the system							
**Atom type**	**A ($kcal^{1/2}$• $mol^{-1/2}$•$Å^6$)**	**B ($kcal^{1/2}$• $mol^{-1/2}$•$Å^3$)**	**C[a] (kcal• mol^{-1})**	**α[a] ($Å^2$)**	**A_{1-4} ($kcal^{1/2}$• $mol^{-1/2}$•$Å^6$)**	**B_{1-4} ($kcal^{1/2}$• $mol^{-1/2}$•$Å^3$)**	**Mass (a.u.)**
H	0.00	0.00			0.00	0.00	1.01
HHM[b]	0.00	0.00			0.00	0.00	1.01
HO	0.00	0.00			0.00	0.00	1.01
HA	69.58	4.91			49.20	3.47	1.01
HC	84.57	5.41			59.80	3.83	1.01
CT	944.52	22.03			667.88	15.58	12.01
CA	1059.13	23.67			748.92	16.74	12.01
CT[b]	944.52	22.03			667.88	15.58	12.01
C2	1802.24	34.18	91.00	2.50	1274.38	24.17	12.01
NO	816.43	23.78			577.31	16.82	14.01
OET	601.15	22.26	53.00	2.50	425.08	15.74	16.00
OP[b]	601.15	22.26	53.00	2.50	425.08	15.74	16.00
OP[c]	445.13	18.25	90.00	2.50	314.76	12.91	16.00
O	616.44	23.77			435.89	16.81	16.00
XO1	976.93	31.26	90.00	2.50	690.79	22.10	16.00
ON	554.63	21.39			392.12	15.12	16.00
O1	616.44	23.77			435.89	16.81	16.00
OHY	690.37	23.86	53.00	2.50	488.17	16.87	16.00
O2Z	873.90	27.96			617.94	19.77	16.00
OHM	956.00	23.01	90.00	2.50	675.99	16.27	16.00
O-	976.93	31.26	90.00	2.50	690.79	22.10	16.00
P	2447.79	46.79	43.00	2.50	1730.85	33.09	30.97

[a] Parameters for the soft pair repulsion potential $V_{soft} = C_i\, C_j\, exp(-\alpha_i\, \alpha_j\, r_{ij})$ that was used to model the VdW interactions only between the atoms involved in bond breaking and bond making processes in the reacting part of the system.
[b] in paraoxon
[c] in *p*-nitrophenyl butyrate

Supplementary Table 4: List of atoms types and corresponding van der Waals parameters used for the atoms in the reacting part of the system.

© The Authors under the terms of the Creative Commons Attribution License http://creativecommons.org/licenses/by/3.0/, which permits unrestricted use, provided the original author and source are credited.

Atom types and partial charges in different VB states									
PXN					EST				
	Atomic charges		VDW types			Atomic charges		VDW types	
#	State I	State II	State I	State II	#	State I	State II	State I	State II
1	1.1976	1.1149	P	P	1	-0.2458	-0.2394	CT	CT
2	-0.6822	-0.7050	O2Z	O2Z	2	0.2066	0.1185	CT	CT
3	-0.4946	-0.4386	OP	OP	3	-0.1422	-0.1811	CT	CT
4	0.4282	0.3878	CT	CT	4	0.7826	0.8205	C2	C2
5	-0.0092	0.0019	HC	HC	5	-0.5587	-0.6241	O	O1
6	-0.0092	0.0019	HC	HC	6	-0.4682	-0.7429	OP	XO1
7	-0.3318	-0.3593	CT	CT	7	0.3931	0.7819	CA	CA
8	0.0918	0.0977	HC	HC	8	-0.2134	-0.4800	CA	CA
9	0.0918	0.0977	HC	HC	9	-0.1959	-0.0814	CA	CA
10	0.0918	0.0977	HC	HC	10	0.0260	-0.1556	CA	CA
11	-0.4946	-0.4386	OP	OP	11	-0.1959	-0.0814	CA	CA
12	0.4282	0.3878	CT	CT	12	-0.2134	-0.4800	CA	CA
13	-0.0092	0.0019	HC	HC	13	0.8038	0.7878	NO	NO
14	-0.0092	0.0019	HC	HC	14	-0.4678	-0.5678	ON	ON
15	-0.3318	-0.3593	CT	CT	15	-0.4678	-0.5678	ON	ON
16	0.0918	0.0977	HC	HC	16	0.0592	0.0585	HC	HC
17	0.0918	0.0977	HC	HC	17	0.0592	0.0585	HC	HC
18	0.0918	0.0977	HC	HC	18	-0.0214	0.0053	HC	HC
19	-0.4664	-0.7441	OP	O-	19	-0.0214	0.0053	HC	HC
20	0.4339	0.7837	CA	CA	20	0.0280	0.0757	HC	HC
21	-0.2471	-0.4779	CA	CA	21	0.0280	0.0757	HC	HC
22	-0.2471	-0.4779	CA	CA	22	0.1799	0.1453	HA	HA
23	-0.1556	-0.0823	CA	CA	23	0.2032	0.1483	HA	HA
24	-0.1556	-0.0823	CA	CA	24	0.2032	0.1483	HA	HA
25	-0.0150	-0.1644	CA	CA	25	0.1799	0.1453	HA	HA
26	0.1843	0.1496	HA	HA	26	0.0592	0.0585	HC	HC
27	0.1843	0.1496	HA	HA	27	-1.1000	-0.7038	OHM	OET
28	0.1940	0.1444	HA	HA	28	0.1000	0.4719	HHM	HO
29	0.1940	0.1444	HA	HA					
30	0.8110	0.7991	NO	NO					
31	-0.4740	-0.5709	ON	ON					
32	-0.4740	-0.5709	ON	ON					
33	-1.1000	-0.6623	OHM	OHY					
34	0.1000	0.4787	HHM	H					

Supplementary Table 5: Atom types and partial charges assigned to atoms in the reacting part of the system in the VB states presented in **Supplementary Figure 1**.

© The Authors under the terms of the Creative Commons Attribution License http://creativecommons.org/licenses/by/3.0/, which permits unrestricted use, provided the original author and source are credited.

Bond stretch parameters in the reacting part of the system							
**Bond type**		**K_b (kcal•mol^{-1}•Å^{-2})**	**r_0 (Å)**	**D_e (kcal•mol^{-1})**	**α (Å^{-2})**	**r_0 (Å)**
C2	CT	634.00	1.52	-	-	-
C2	O	1140.00	1.23	-	-	-
C2	OP	428.00	1.33	-	-	-
CA	CA	938.00	1.40	-	-	-
CA	HA	734.00	1.08	-	-	-
CA	NO	800.00	1.46	-	-	-
CA	XO1	900.00	1.36	-	-	-
CA	OP	900.00	1.36	-	-	-
CT	CT	536.00	1.53	-	-	-
CT	OP	640.00	1.41	-	-	-
HC	CT	680.00	1.09	-	-	-
NO	ON	1100.00	1.23	-	-	-
OHM	HHM	1106.00	0.96	-	-	-
P	O2Z	1050.00	1.48	-	-	-
P	OP	460.00	1.61	-	-	-
C2	OP	-	-	93.00	2.00	1.25
C2	OET	-	-	93.00	2.00	1.25
P	OP	-	-	95.00	2.00	1.61
P	OHY	-	-	95.00	2.00	1.61

Supplementary Table 6: Bond parameters used for the reacting part of the system. Bond stretch potential energy was described using: Harmonic potential: $V_{Harmonic} = 0.5\ K_b(r-r_0)^2$ and Morse potential: $V_{Morse} = D_e\ (1 - e^{-\alpha(r-r_0)})^2$.

© The Authors under the terms of the Creative Commons Attribution License http://creativecommons.org/licenses/by/3.0/, which permits unrestricted use, provided the original author and source are credited.

**Angle bend parameters in the reacting part of the system**				
	**Angle type**		**K_a (kcal•mol^{-1}•rad^{-2})**	**Θ_0 (°)**
C2	OET	HO	70.00	113.00
C2	CT	CT	126.00	111.10
C2	OP	CA	166.00	116.90
CA	CA	CA	126.00	120.00
CA	CA	HA	70.00	120.00
CA	CA	NO	170.00	120.00
CA	CA	OP	140.00	120.00
CA	CA	XO1	140.00	120.00
CA	NO	ON	160.00	117.50
CA	OP	P	100.00	120.50
CT	C2	OET	140.00	108.00
CT	C2	O	160.00	120.40
CT	C2	OP	162.00	111.40
CT	CT	CT	116.70	112.70
CT	CT	OP	100.00	109.50
CT	OP	P	100.00	120.50
HC	CT	C2	70.00	109.50
HC	CT	CT	75.00	110.70
HC	CT	HC	66.00	107.80
HC	CT	OP	70.00	109.50
O	C2	OP	166.00	123.40
O1	C2	OET	160.00	121.00
O2Z	P	OP	200.00	108.23
OHY	P	O2Z	200.00	108.23
OHY	P	OP	200.00	108.23
ON	NO	ON	160.00	125.00
OP	P	OP	90.00	102.60
P	OHY	H	90.00	108.50

Supplementary Table 7: Angle parameters used for the reacting part of the system. Angle bend potential energy function: $V_{Angle} = K_a(\Theta-\Theta_0)^2$.

© The Authors under the terms of the Creative Commons Attribution License http://creativecommons.org/licenses/by/3.0/, which permits unrestricted use, provided the original author and source are credited.

Torsion parameters in the reacting part of the system						
**Torsion type**				**V_1 (kcal•mol^{-1})**	**V_2 (kcal•mol^{-1})**	**V_3 (kcal•mol^{-1})**
C2	OP	CA	CA	0.000	1.250	0.000
CA	CA	NO	ON	0.000	0.575	0.000
CA	CA	OP	P	0.000	1.495	0.000
CA	CA	CA	CA	0.000	3.625	0.000
CA	CA	CA	HA	0.000	3.625	0.000
CA	CA	CA	NO	0.000	3.625	0.000
CA	CA	CA	OP	0.000	3.625	0.000
CA	CA	CA	XO1	0.000	3.625	0.000
CA	OP	P	O2Z	0.000	0.000	0.281
CA	OP	P	OP	0.000	0.000	0.281
CT	CT	C2	OP	0.000	0.000	-0.277
CT	CT	C2	O1	0.000	0.273	0.000
CT	CT	C2	O	-0.138	0.614	-0.347
CT	CT	OP	P	0.000	1.495	0.000
CT	C2	OP	CA	0.750	2.500	0.000
CT	C2	OH	HA	0.750	2.750	0.000
CT	CT	CT	HC	0.000	0.000	0.150
CT	CT	CT	C2 [a]	-0.848	-0.228	0.292
CT	CT	CT	C2 [b]	-1.030	-0.157	0.158
CT	CT	C2	OET	0.500	0.273	0.225
CT	OP	P	O2Z	0.000	0.000	0.281
CT	OP	P	OP	0.000	0.000	0.281
HA	CA	CA	HA	0.000	3.625	0.000
HA	CA	CA	NO	0.000	3.625	0.000
HA	CA	CA	OP	0.000	3.625	0.000
HA	CA	CA	XO1	0.000	3.625	0.000
HC	CT	CT	C2 [a]	0.000	0.000	-0.038
HC	CT	CT	C2 [b]	0.000	0.000	-0.050
HC	CT	C2	O	0.000	0.000	0.000
HC	CT	C2	OET	0.000	0.000	0.000
HC	CT	C2	O1	0.000	0.000	0.000
HC	CT	C2	OP	0.000	0.000	0.066
HC	CT	CT	HC	0.000	0.000	0.150
HC	CT	CT	OP	0.000	0.000	0.234
HO	OET	C2	O1	0.000	2.750	0.000
HO	OHY	P	O2Z	0.000	0.000	0.281
HO	OHY	P	OP	0.000	0.000	0.281
O	C2	OP	CA	0.000	2.500	0.000
OHY	P	OP	CT	0.000	0.000	0.281
P	OP	CT	HC	0.000	0.000	0.179

[a] EVB State I
[b] EVB State II

Supplementary Table 8: Torsion parameters used for the reacting part of the system. Torsion potential energy function: $V_{Torsion} = V_1(1+\cos \Phi) + V_2(1-\cos 2\Phi) + V_3(1+\cos 3\Phi)$.

© The Authors under the terms of the Creative Commons Attribution License http://creativecommons.org/licenses/by/3.0/, which permits unrestricted use, provided the original author and source are credited.

Improper torsion parameters in the reacting part of the system					
Improper torsion type				K_ξ (kcal•mol^{-1}•rad^{-2})	ξ_0 (°)
CA	CA	CA	XO1	1.10	180.00
CA	CA	CA	OP	1.10	180.00
CA	NO	ON	ON	10.50	180.00
CT	C2	O	OP	10.50	180.00
CT	C2	O1	OET	10.50	180.00
HA	CA	CA	CA	1.10	180.00
NO	CA	CA	CA	1.10	180.00

Supplementary Table 9: Improper torsion parameters used for the reacting part of the system. Improper torsion potential energy function: $V_{Improper} = K_\xi (\xi-\xi_0)^2$.

Substrate	H_{ij} (kcal•mol^{-1})	α_{ij} (kcal•mol^{-1})
PXN	89.0	-26.3
EST	54.7	-27.3

Supplementary Table 10: EVB parameters used in this work. H_{ij} is the off-diagonal term in the EVB Hamiltonian, while α_{ij} is the energy difference between the two valence states in the reference reaction. Both parameters were fitted to reproduce activation and reaction free energies of the reference reactions.

References

[1] Barrozo, A., Duarte, F., Bauer, P., Carvalho, A.T. & Kamerlin, S.C.L. 2015 Cooperative electrostatic interactions drive functional evolution in the alkaline phosphatase superfamily. *J. Am. Chem. Soc.* **137**, 9061-9076.

[2] Ben-David, M., Sussman, J.L., Maxwell, C.I., Szeler, K., Kamerlin, S.C. & Tawfik, D.S. 2015 Catalytic stimulation by restrained active-site floppiness--the case of high density lipoprotein-bound serum paraoxonase-1. *J. Mol. Biol.* **427**, 1359-1374.

[3] Åqvist, J. & Kamerlin, S.C.L. 2016 Conserved motifs in different classes of GTPases dictate their specific modes of catalysis. *ACS Catal.* **6**, 1737-1743.

[4] Warshel, A. & Weiss, R.M. 1980 An empirical valence bond approach for comparing reactions in solutions and in enzymes. *J. Am. Chem. Soc.* **102**, 6218-6226.

[5] Warshel, A. 1991 *Computer modeling of chemical reactions in enzymes and solutions.* New York, Wiley.

[6] Shurki, A., Derat, E., Barrozo, A. & Kamerlin, S.C.L. 2015 How valence bond theory can help you understand your (bio)chemical reaction. *Chem. Soc. Rev.* **44**, 1037-1052.

[7] Warshel, A., Sharma, P.K., Kato, M., Xiang, Y., Liu, H. & Olsson, M.H.M. 2006 Electrostatic basis for enzyme catalysis. *Chem. Rev.* **106**, 3210-3235.

© The Authors under the terms of the Creative Commons Attribution License http://creativecommons.org/licenses/by/3.0/, which permits unrestricted use, provided the original author and source are credited.

[8] Berman, H.M., Westbrook, J., Feng, Z., Guilliland, G., Bhat, T.N., Weissig, H., Shindyalov, I.N. & Bourne, P.E. 2000 The Protein Data Bank. *Nucleic Acids Res.* **28**, 235-242.

[9] Rose, P.W., Prlic, A., Bi, C., Bluhm, W.F., Christie, C.H., Dutta, S., Green, R.K., Goodsell, D.S., Westbrook, J.D., Woo, J., et al. 2015 The RCSB Protein Data Bank: Views of structural biology for basic and applied research and education. *Nucleic Acids Res.* **43**, D345-D356.

[10] Dong, Y.J., Bartlam, L., Sun, Y.F., Zhou, Z.P., Zhang, C.G., Zhang, Z., Rao, Z. & Zhang, X.E. 2005 Crystal structure of methyl parathion hydrolase from *Pseudomonas sp.* WBC-3. *J. Mol. Biol.* **353**, 655-663.

[11] Jackson, C.J., Foo, J.L., Kim, H.-K., Carr, P.D., Liu, J.W., Salem, G. & Ollis, D.L. 2008 In crystallo capture of a Michaelis complex and product binding modes of a bacterial phosphotriesterase. *J. Mol. Biol.* **375**, 1189-1196.

[12] Marelius, J., Kolmodin, K., Feierberg, I. & Aqvist, J. 1998 Q: A molecular dynamics program for free energy calculations and empirical valence bond simulations in biomolecular systems. *J. Mol. Graph. Mod.* **16**, 213-225.

[13] Jorgensen, W.L., Maxwell, D.S. & TiradoRives, J. 1996 Development and testing of the OPLS all-atom force field on conformational energetics and properties of organic liquids. *J. Am. Chem. Soc.* **118**, 11225-11236.

[14] Schrödinger, L. 2014 Schrödinger Release 2014-1: MacroModel, Version 10.3. (New York, NY.

[15] Cieplak, P., Cornell, W.D., Bayly, C. & Kollman, P.A. 1995 Application of the multimolecule and multiconformational RESP methodology to biopolymers - Charge derivation for DNA, RNA, and proteins. *J. Comp. Chem.* **16**, 1357-1377.

[16] Wang, J.M., Wang, W., Kollman, P.A. & Case, D.A. 2006 Automatic atom type and bond type perception in molecular mechanical calculations. *J. Mol. Graph. Mod.* **25**, 247-260.

[17] Case, D.A., Darden, T.A., Cheatham III, T.E., Simmerling, C.L., Wang, J., Duke, R.E., Luo, R., Walker, R.C., Zhang, W., Merz, K.M., et al. 2012 AMBER 12. (San Franciso, University of California.

[18] Frisch, M.J., Trucks, G.W., Schlegel, H.B., Scuseria, G.E., Robb, M.A., Cheeseman, J.R., Scalmani, G., Barone, V., Mennucci, B., Petersson, G.A., et al. 2009 Gaussian 09, Rev C01. Wallingford, CT, USA, Gaussian, Inc.

[19] King, G. & Warshel, A. 1989 A surface constrained all-atom solvent model for effective simulations of polar solutions. *J. Chem. Phys.* **91**, 3647-3661.

[20] Jorgensen, W.L., Chandrasekhar, J., Madura, J.D., Impey, R.W. & Klein, M.L. 1983 Comparison of simple potential functions for simulating liquid water. *J. Chem. Phys.* **79**, 926-935.

[21] Amrein, B.A., Bauer, P., Duarte, F., Janfalk Carlsson, A., Naworyta, A., Mowbray, S.L., Widersten, M. & Kamerlin, S.C.L. 2015 Expanding the catalytic triad in epoxide hydrolases and related enzymes. *ACS Catal.* **5**, 5702-5713.

[22] Ryckaert, J.P., Ciccotti, G. & Berendsen, H.J.C. 1977 Numerical-integration of cartesian equations of motion of a system with constraints - Molecular-dynamics of N-alkanes. *J. Comp. Phys.* **23**, 327-341.

© The Authors under the terms of the Creative Commons Attribution License http://creativecommons.org/licenses/by/3.0/, which permits unrestricted use, provided the original author and source are credited.

[23] Sondergaard, C.R., Olsson, M.H., Rostkowski, M. & Jensen, J.H. 2011 Improved treatment of ligands and coupling effects in empirical calculation and rationalization of pKa values. *J. Chem. Theory Comput.* **7**, 2284-2295.

[24] Olsson, M.H., Sondergaard, C.R., Rostkowski, M. & Jensen, J.H. 2011 PROPKA3: Consistent treatment of internal and surface residues in empirical pKa predictions. *J. Chem. Theory Comput.* **7**, 525-537.

[25] Åqvist, J. & Warshel, A. 1989 Calculations of free energy profiles for the staphylococcal nuclease catalyzed reaction. *Biochemistry* **28**, 4680-4689.

[26] Duarte, F., Bauer, P., Barrozo, A., Amrein, B.A., Purg, M., Aqvist, J. & Kamerlin, S.C.L. 2014 Force field independent metal parameters using a nonbonded dummy model. *J. Phys. Chem. B* **118**, 4351-4362.

[27] Bürgi, H.B., Dunitz, J.D., Lehn, J.M. & Wipff, G. 1974 Stereochemistry of reaction paths at carbonyl centres. *Tetrahedron* **30**, 1563-1572.

[28] Lee, F.S. & Warshel, A. 1992 A local reaction field method for fast evaluation of long-range electrostatic interactions in molecular simulations. *J. Chem. Phys.* **97**, 3100-3107.

[29] Berendsen, H.J.C., Postma, J.P.M., Vangunsteren, W.F., Dinola, A. & Haak, J.R. 1984 Molecular-dynamics with coupling to an external bath. *J. Chem. Phys.* **81**, 3684-3690.

[30] Purcell, J. & Hengge, A.C. 2005 The thermodynamics of phosphate versus phosphorothioate ester hydrolysis. *J. Org. Chem.* **70**, 8437-8442.

[31] Caldwell, S.R. & Raushel, F. 1991 Primary and secondary oxygen-18 isotope effects in the alkaline and enzyme-catalyzed hydrolysis of phosphotriesters. *J. Am. Chem. Soc.* **113**, 730-732.

[32] Caldwell, S.R., Raushel, F.M., Weiss, P.M. & Cleland, W.W. 1991 Transition-state structures for enzymatic and alkaline phosphotriester hydrolysis. *Biochemistry* **30**, 7444-7450.

[33] Matta, M.S. & Toenjes, A.A. 1985 Solvation effects on the alkaline hydrolysis of some *p*-nitrophenyl esters. *J. Am. Chem. Soc.* **7591-7596**.

[34] Åqvist, J. & Kamerlin, S.C.L. 2015 The conformation of a catalytic loop is essential to GTP hydrolysis on the ribosome. *Biochemistry* **54**, 546–556.

[35] Baier, F., Chen, J., Solomonson, M., Strynadka, N.C. & Tokuriki, N. 2015 Distinct metal isoforms underlie promiscuous activity profiles of metalloenzymes. *ACS Chem. Biol.* **10**, 1684-1693.

© The Authors under the terms of the Creative Commons Attribution License http://creativecommons.org/licenses/by/3.0/, which permits unrestricted use, provided the original author and source are credited.
